# Right Ventricular Apical (RVA) Pacing-Induced Cardiomyopathy Disguised As Ischemic Cardiomyopathy: A Case Report

**DOI:** 10.7759/cureus.91563

**Published:** 2025-09-03

**Authors:** Ridhika Prasad, Vinh Nguyen, Robert Tungate, Michael Rochon-Duck, Anil K Tiwari

**Affiliations:** 1 School of Medicine, Burrell College of Osteopathic Medicine, University Park, USA; 2 Anesthesiology and Perioperative Medicine, California University of Science and Medicine, Riverside, USA; 3 Cardiology, UCI Health, Orange, USA; 4 Anesthesiology and Perioperative Medicine, UCI Health, Orange, USA

**Keywords:** and transesophageal echocardiography, biventricular pacing, ejection fraction, hypokinesis, lv pacing, rv pacing

## Abstract

Cardiac pacing is an effective treatment for patients with symptomatic bradycardia. The majority of patients tolerate right ventricular apical (RVA) pacing, which remains the standard of care for patients who have a normal ejection fraction (EF). However, a significant minority experience adverse left ventricular (LV) remodeling and a decline in EF. In this case report, we describe a patient with chronic RVA pacing and an EF of 20% with hypokinesis of mid-septal and inferior wall segments who was scheduled for mitral valve replacement, Cox-Maze, left atrial appendage clipping, and possible coronary artery bypass graft (CABG). After bi-ventricular pacing, EF improved to 45% due to improved function of the mid-septal and inferior wall segments.

## Introduction

Cardiac pacing is an effective treatment for patients with symptomatic bradycardia. The majority of patients tolerate right ventricular apical (RVA) pacing, which remains the standard of care for patients who have a normal ejection fraction (EF). However, a significant minority experience adverse left ventricular (LV) remodeling and a decline in ejection fraction. 10% to 20% of patients with frequent conventional RV pacing develop pacemaker-induced cardiomyopathy. Contributors to risk include male sex, wider paced QRS durations, and higher burden of RV pacing, especially over 40%. A paced QRS length ≥163 ms notably increases the risk of heart failure hospitalization. RV pacing depolarizes the right ventricle first, then the left ventricle second. This causes dyssynchronous contraction, similar to a left bundle branch block (LBBB) pattern as seen in our patient. In this case report, we describe a patient with chronic RVA pacing and an EF of 20% with hypokinesis of mid-septal and inferior wall segments who was scheduled for mitral valve replacement, Cox-Maze, and left atrial appendage clipping.

## Case presentation

We present a case of a 62-year-old male patient (72 kilograms, 175 centimeters, Body Mass Index 23.5 kg/m^2^), with a past medical history of severe mitral regurgitation, moderate alcohol use disorder, atrial fibrillation, and decompensated heart failure. Four years prior to this admission, he received an Epic™ bioprosthetic 33 mm mitral valve (Abbott Laboratories, Abbott Park, United States) and a Boston Scientific single-chamber implantable cardioverter defibrillator (ICD) (Boston Scientific Corporation, Marlborough, United States). 

**Figure 1:**
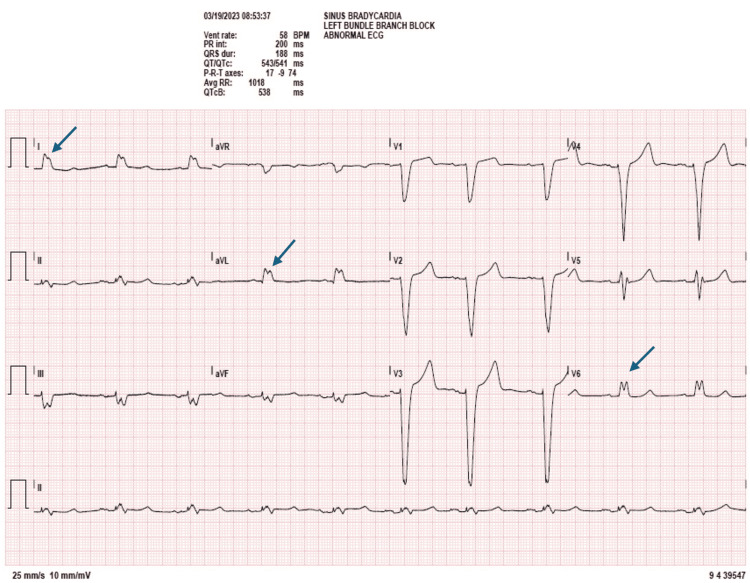
Preoperative EKG (not paced) Arrows pointing at left bundle branch block (LBBB) on the EKG.

**Figure 2:**
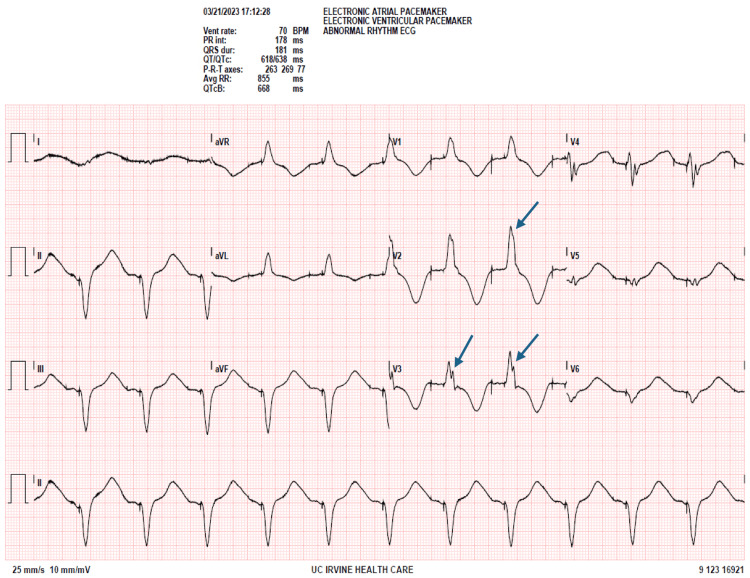
Preoperative EKG (paced) Right ventricular paced rhythm set at 70 beats per minute. Arrows point at left bundle branch block (LBBB).

Preoperatively, a transthoracic echocardiogram (TTE) showed a left ventricular ejection fraction (LVEF) of 20%, moderately increased LV internal size, mild concentric LV hypertrophy, mild dilation of the left atrium, mild pulmonary hypertension (HTN) with right ventricular systolic pressure (RVSP) of 42 mmHg, and trace tricuspid regurgitation without vegetation. Heart catheterization showed normal coronary arteries, ruling out ischemic cardiomyopathy. EKG revealed sinus bradycardia in addition to a left bundle branch block with a QRS duration of 188 ms (Figure 1). The right ventricular paced rhythm was set at 70 beats per minute (Figure 2). Chest X-ray showed median sternotomy wires and an automatic implantable cardioverter defibrillator (AICD) with lead terminating over the right ventricle.

After work-up, the patient was scheduled for redo sternotomy, mitral valve replacement, possible Cox-Maze with left atrial appendage clipping, and possible coronary artery bypass graft (CABG). 

Intraoperatively, transesophageal echocardiography (TEE) was performed, suggesting a left ventricular ejection fraction of 25%, dyskinesis of the septal walls, and hypokinesis of the inferior walls (Video 1).

**Video 1:** Intraoperative TEE of Left Ventricle with RV pacing TEE: transesophageal echocardiogram; RV: right ventricle

The porcine mitral valve (St Jude Epic 33 mm (Abbott Laboratories, Saint Paul, United States)) was removed and replaced with an Edwards Mitris Resilia bioprosthesis 33 mm (Edwards Lifesciences, Irvine, United States). Cox-Maze procedure performed using cryotherapy, and left atrial appendage clipped using AtriCure size 45 mm (AtriCure, Mason, United States). Given the dyskinesis of the left ventricle as viewed on TEE and direct visualization of the heart, a decision was made to explant the AICD/pacemaker (Boston Scientific RV 0296) and replace it with a Boston Scientific Momentum CRT-D Bi-Ventricular AICD. The American Heart Association, American College of Cardiology, and the Heart Rhythm Society guidelines give a Class 1 recommendation for Cardiac Resynchronization Therapy in patients with LVEF of less than or equal to 35%, QRS greater than or equal to 150 ms with LBBB, and an expected high RV pacing burden.

After bi-ventricular pacing commenced, intraoperative TEE imaging showed an improvement in the mid-septal and inferior wall segments, with EF increasing to 45% (Video 2). Improvements in LVEF occurred immediately after bi-ventricular pacing. The patient was discharged to go home on postoperative day 9.

**Video 2:** Intraoperative TEE of the left ventricle with bi-ventricular pacing showing improved function TEE: transesophageal echocardiography

## Discussion

The deleterious effects of RVA pacing on LV systolic function have been extensively documented [1-4]. Reported causes in the literature include myocardial perfusion defects and pacing-induced LV dyssynchrony [5,6]. These mechanisms have many downstream effects, which include adverse LV remodeling, LV dilatation, LV asymmetrical hypertrophy, and, ultimately, a decline in LVEF [2-6]. Pacing-induced cardiomyopathy is associated with high degree of RV pacing, wider paced QRS duration, and lower baseline LV function [4]. For these reasons, bi-ventricular pacemaker implantation carries a Class I recommendation from the American Heart Association/American College of Cardiology for patients with New York Heart Association Class II or III heart failure with LVEF <35%, a QRS duration >150ms, and a left bundle branch block, regardless of the expected pacing burden.

As technological advancements continue to increase the success rate of left ventricular lead implantation via the coronary sinus branches, bi-ventricular (BiV) pacing becomes a more viable alternative to improve patient outcomes [2]. There is substantial evidence that in patients with left ventricular dysfunction and clinical indications for pacing, such as atrioventricular block, physiologic pacing with BiV devices, left bundle area pacing, or His bundle pacing provides significant clinical benefit over RVA pacing for LV performance and patient outcomes [5, 7]. The mechanism of enhanced cardiac function may be improved cardiac synchrony, improved myocardial perfusion, and minimized myofibril disarray, leading to reverse LV remodeling [8]. These benefits have also been shown to extend to patients with normal EF or no clinical indication for BiV pacing [9].

As noted in this case, upgrading to BiV pacing can help reverse RVA pacing-induced cardiomyopathy in some patients. Previous research has shown that this conversion from RVA to BiV pacing reverses cardiac dyssynchrony, improving LV ventricular performance and dimensions [10-15]. According to the BLOCK HF Study in 2013, in AV block with LVEF ≤ 50%, BiV pacing reduced the composite of death/heart failure (HF) urgent care/adverse remodeling, and improved New York Heart Association class and quality of life versus RV pacing [16]. This leads to a significant increase in EF, which can be comparable to de novo BiV pacing. In this case, the patient’s improvement in EF following LV pacing is due to improvement in the mid-septal and inferior wall segments. In the future, this use of a bi-ventricular device may also allow for a greater tolerance of a high ventricular pacing burden, permitting more aggressive pharmacologic suppression of atrial fibrillation. 

## Conclusions

This case report demonstrates the potential benefits of physiologic bi-ventricular pacing for patients undergoing cardiac surgery with low EF, widened QRS complex, and RV pacing-induced cardiomyopathy. Although current evidence has established the detrimental effects of chronic right ventricular apical pacing and the therapeutic role of cardiac resynchronization therapy, once pacing-induced cardiomyopathy has developed, the role of preventative strategies in patients at risk remains less clearly defined. Bi-ventricular pacing is selected as the therapeutic strategy for pacing-induced cardiomyopathy because it is the only modality with robust mechanistic rationale, randomized trial evidence, and guideline endorsement demonstrating reverse remodeling, improved functional status, and survival benefit in patients with pacing-induced dyssynchrony.

While conduction system pacing (His bundle or left bundle branch area pacing) holds promise for prevention, bi-ventricular pacing remains the gold standard therapy once pacing-induced cardiomyopathy develops. Although findings from a single case do not establish causality, closer monitoring of patients with RV pacing is recommended, and early intervention with LV pacing may prevent the development of cardiomyopathy and improve cardiac function overall.
